# Urethral Plate Characteristics in Cases of Non-proximal Hypospadias May Not Be Associated With a Higher Risk of Complications When a Two-Stage Repair Is Applied

**DOI:** 10.3389/fped.2022.900514

**Published:** 2022-07-08

**Authors:** Marios Marcou, Sarah-Magdalena Bobbe, Bernd Wullich, Karin Hirsch-Koch

**Affiliations:** ^1^Clinic of Urology and Pediatric Urology, University Hospital Erlangen, Erlangen, Germany; ^2^Clinic of Urology, Dr. Lubos Kliniken Bogenhausen, Munich, Germany

**Keywords:** children, hypospadias repair, distal hypospadias, two-stage repair, Thiersch-Duplay, non-proximal hypospadias, mid-shaft hypospadias, urethral plate

## Abstract

**Purpose:**

To investigate whether a two-stage repair of distal- and mid-shaft hypospadias (non-proximal hypospadias) could eliminate the risk factors resulting from adverse urethral plate characteristics and eventually reduce complication rates.

**Methods:**

We retrospectively reviewed all cases of primary surgical repair of non-proximal hypospadias performed in our center between 2009 and 2018. In all cases where adverse urethral plate characteristics were found, such as meatal stenosis, a shallow urethral groove, a thick web of tissue between the native meatus and the urethral groove or in the presence of a very “thin,” skin-like distal urethra, a two-stage repair was routinely undertaken. In cases of native meatal stenosis, a meatotomy, and meatoplasty were performed. In cases of a very “thin” distal urethra we incised the skin proximally up to the point of a normal urethral fold and a meatoplasty was performed at that point. Hypospadias repair was then performed in a second operation, 3–6 months following the first procedure. Urethroplasty, both in cases of a single-stage repair and in cases of a two-stage repair, was always performed using the Thiersch-Duplay technique. Patients with a follow-up of less than 12 months were excluded from this study.

**Results:**

Over a period of 10 years, 208 boys underwent primary surgical repair of non-proximal hypospadias. Eighty-nine of the 208 patients (42.8%) underwent single-stage hypospadias repair. Two-stage repair of the hypospadias was required in 119 (57.2%) of the patients. The overall complication rate was 3.4% in the group operated in a single stage and 7.6% in the group that required a two-stage repair (*p* = 0.09). The most frequent complication reported was urethrocutaneous fistula (*p* = 0.31), followed by meatal stenosis (*p* = 0.37), urethral stricture (*p* = 0.08) and wound dehiscence (*p* = 0.16). There was no significant difference between the complication rates of the two groups.

**Conclusion:**

Patients with distal hypospadias and poor urethral plate characteristics repaired in a two-stage approach have comparable low-complications to those with favorable urethral plate characteristics repaired in a single-stage.

## Introduction

More than 300 surgical procedures and modifications have been proposed for hypospadias repair, with some surgeons stating with amusement that there are almost as many techniques for repairing hypospadias as there are surgeons who perform the procedure (1). Repair of hypospadias through tubularization of an intact urethral plate, first described by Thiersch and Duplay, has remained one of the most popular methods of hypospadias repair for more than 150 years. The Thiersch-Duplay technique offers several advantages. It utilizes the tissues that were embryonically destined to form a urethra, there is minimal disturbance to the blood supply of the urethral plate, which is often well vascularized, and there is hardly any contracture of the tubularized plates (2).

Although hypospadias is usually defined by the position of the ectopic meatus, a wide spectrum of anatomical variations has been described, and complications have repeatedly been associated with urethral plate characteristics (3–7). The Thiersch-Duplay method can ideally be performed on a hypospadias patient with a wide urethral plate and a deep urethral groove (8). Unfortunately, in approximately 50% of cases, there is meatal stenosis and a shallow groove (9); therefore, in order that the Thiersch-Duplay technique can be applied, meatoplasty or an incision of the urethral plate is required prior to tubularization (9, 10). Some studies have reported that with the use of the tubularized incised plate technique (TIP-Technique), a modification of the Thiersch-Duplay technique, urethral plate characteristics do not correlate with urethroplasty complications (11).

Following meatoplasty or an incision of the urethral plate, the urethral groove is deepened, and the widened neomeatus is transferred to a deeper level (9). To date, in cases of non-proximal hypospadias, meatoplasty or incision of the urethral plate followed by tubularization of the urethral plate, has always been performed as a single-stage procedure. Although there is growing evidence that a two-stage repair in cases of proximal hypospadias could be associated with fewer complications when compared to single-stage repairs (12, 13), to our knowledge, a two-stage repair has never been routinely applied in cases of non-proximal hypospadias. With this study, we therefore review all cases of primary surgical repair of distal and mid-shaft hypospadias in our center between 2009 and 2018 to ask whether a two-stage repair could eliminate the risk factors resulting from adverse urethral plate characteristics and eventually reduce complication rates in the surgical repair of non-proximal hypospadias.

## Materials and Methods

We retrospectively reviewed all cases of primary surgical repair of distal and mid-shaft hypospadias (non-proximal hypospadias) that were performed in our single-center between 2009 and 2018, through five specialized surgeons. During this period in our center, in all primary cases of non-proximal hypospadias, where a straightforward tubularization of the urethra was intraoperatively deemed not possible, a two-stage approach in the primary repair was routinely applied.

**FIGURE 1:**
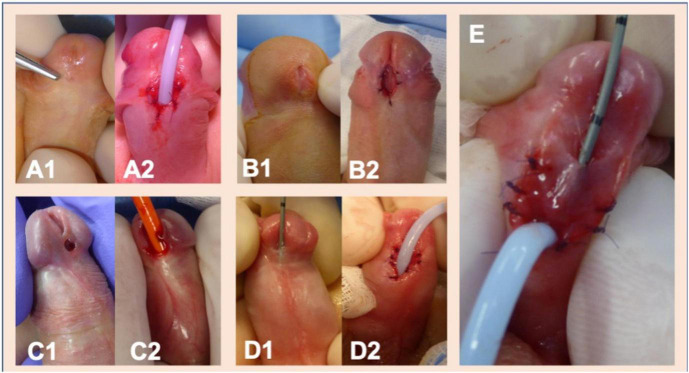
The polymorphism of distal and mid-shaft hypospadias and some examples of indications for two-stage hypospadias repair in our center. **(A1,A2)** Meatal stenosis and a shallow glanular groove in a case of coronal hypospadias. **(B1,B2)** Meatotomy in a case of coronal hypospadias reveals a blind-ending urethral duct extending distally underneath the shallow urethral groove. A complete incision of the ventral wall of the blind-ending urethral duct was performed. **(C1,C2)** A thick web of tissue separates the hypospadic meatus with the urethral pit. **(D1,D2)** An example of a very “thin” distal urethra that cannot be separated from the overlying skin. Following incision of the very “thin” urethra to the point of healthy urethral tissue, meatoplasty was then performed at this point. **(E)** An example of how various characteristics are combined. Following the incision of a very “thin” skinlike urethra, urethral duplication with a deep blind ending urethral channel parallel to the urethra is revealed. Unification of the blind ending accessory urethral channel with the urethra through resection of the separating tissue was undertaken.

**FIGURE 2:**
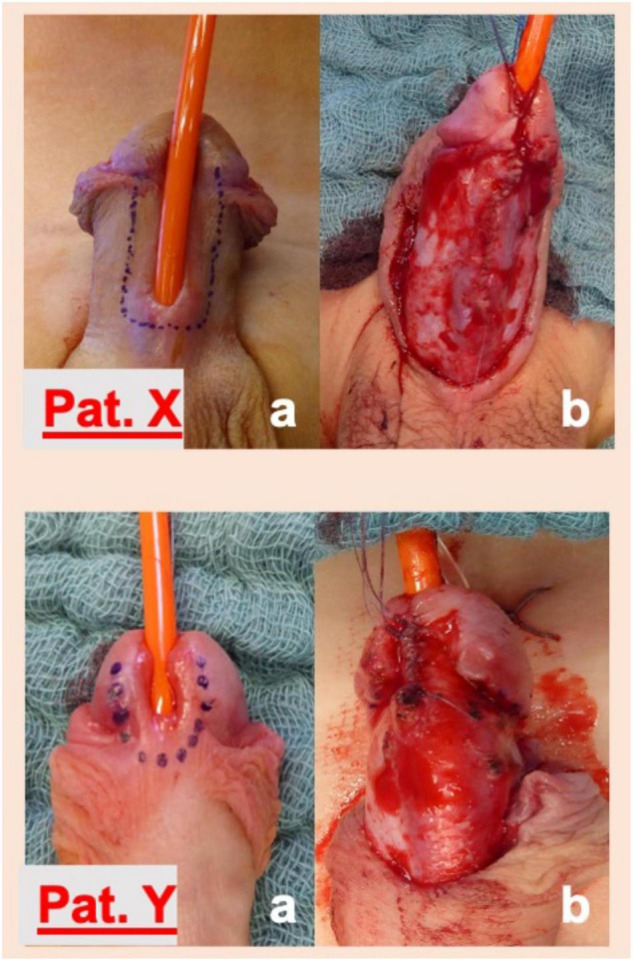
Formation of the neourethra in patients with a mid-shaft (Pat. X) and a coronal (Pat. Y) hypospadias. Following a U-shaped incision around the native hypospadic meatus and along the lateral margins of the urethral plate (a), tension-free tubularization of the laterally augmented urethral plate around a 10 Fr. catheter using a 6.0 monocryl running suture in the Thiersch-Duplay technique was performed. In cases of a narrow urethral plate the vertical parallel incisions were made 2–3 mm lateral to the border of the urethral plate. This maneuver adds 4–6 mm to the width of the urethral plate and allows tension free tubularization of the plate. The suture was covered thereafter with paraurethral tissue using a 6.0 monocryl running suture (not illustrated here).

**FIGURE 3:**
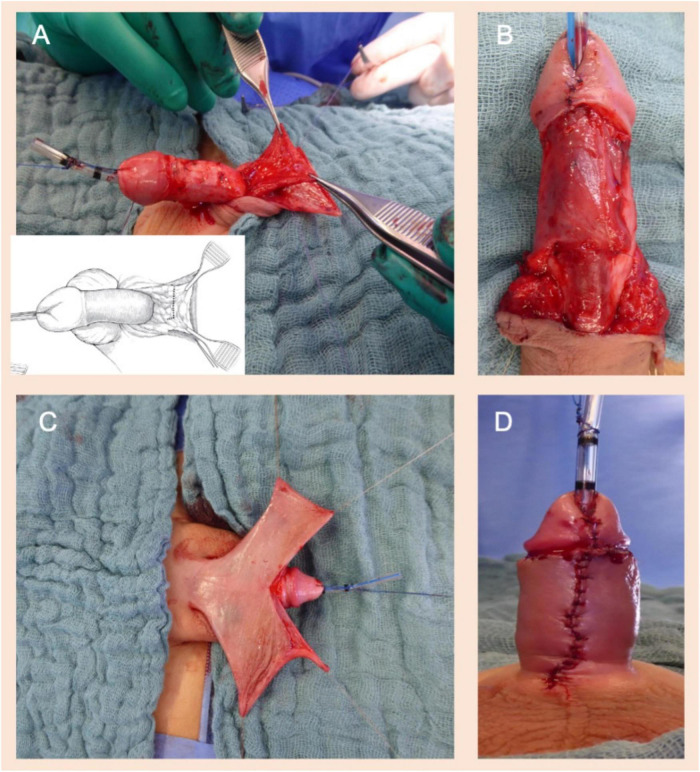
**(A)** Harvesting a pedicled dartos flap from the excess preputial hood. **(B)** The pedicled dartos flap is rotated from the dorsal to the ventral side of the penis to form an extra layer of coverage for the neourethra. Extra care is provided that the fixation of the dartos flap, laterally to the neo-urethra, would be tension-free to avoid rotation of the penis. **(C)** In cases of ventral skin paucity, symmetrical dorsal skin flaps are rotated to the ventral side of the penis and used to cover the ventral penile shaft (modified Byars’ flaps). **(D)** The penile raphe was imitated by creating a midline ventral suture line. A compression dressing of the penis was placed and left for 5 days (not illustrated here). The catheter was removed 7–10 days after the operation.

In cases of non-proximal hypospadias where adverse urethral plate characteristics were found, such as meatal stenosis, defined as a meatal caliber of less than 8 Fr (6, 7), a shallow urethral groove (5, 6), a thick web of tissue between the native meatus and the urethral groove or in the presence of a very “thin” distal urethra that could not be separated from the overlying shaft skin (14), a two-stage repair was routinely undertaken (Figure 1). Following a first-stage operation that would ultimately lead to a healthy distal urethra with a wide meatus (Figure 1), urethroplasty using the Thiersch-Duplay technique was then performed in a second procedure 3–6 months later. In cases of native meatal stenosis, a meatotomy and meatoplasty were performed. In cases of a very “thin” distal urethra we incised the skin proximally up to the point of a normal urethral fold and a meatoplasty was performed at that point. Hypospadias repair was then performed in a second operation, 3–6 months following the first procedure. Urethroplasty, both in cases of a single-stage repair and in cases of a two-stage repair, was always performed using the Thiersch-Duplay technique, as described in Figures 2, 3. In cases of a narrow urethral plate lateral augmentation of the urethral plate was undertaken in both groups (Figure 2). No other techniques in the repair of non-proximal hypospadias were used in this period. Urethroplasty was performed identically in both groups and there was no difference in the type of suture used, the suture technique, the applied dressing type as well as the size and type of the catheter used.

Follow-up visits were scheduled in the outpatient department of our clinic at 1 week, 6 weeks, and 6 months postoperatively and once per year thereafter. Follow-up was defined as the time between the urethroplasty and the last visit of the patient in our outpatient department. Patients with a follow-up of less than 12 months were excluded from this study. The clinical data were statistically evaluated using an unpaired two-sample *t*-test and a Mann–Whitney *U*-test. Statistical significance was set at *p* < 0.05.

## Results

Over a period of 10 years, 228 boys with non-proximal hypospadias underwent primary surgical repair in our clinic. None of the children received testosterone therapy prior to the surgical repair. Twenty children with a follow-up of less than 12 months were excluded from this study. The median age of the remaining 208 boys was 18 months (IQR 13–40 months) at the time of urethroplasty. Eighty-nine of the 208 patients (42.8%) underwent single-stage hypospadias repair. In 119 patients (57.2%), a two-stage repair of the hypospadias was undertaken. The median follow-up of the 208 patients was 50 months (IQR 30–75 months). Overall complications were reported in 12 of the 208 patients (5.8%), without a significant difference between the two groups (*p* = 0.09, *t*-test) (Table 1).

**TABLE 1:** Comparison of the complication rates following non-proximal hypospadias repair between the patient groups that underwent single-stage and two-stage procedures.

	Single-stage procedure (*n* = 89)	Two-stage procedure (*n* = 119)	*T*-test
Median age of the patients (at time of the 1. operation)	20 months	17 months	*P* = 0,08
Median follow-up	49 months	53 months	*P* = 0,32
Median time to complication	27 months	12 months	*p* = 0.2
**Complications reported:**			
Urethrocutaneous fistula	2.3% (*n* = 2)	3.4% (*n* = 4)	*P* = 0.31
Meatal stenosis	1.1% (*n* = 1)	1.7% (*n* = 2)	*P* = 0.37
Urethral stricture	0%	1.7% (*n* = 2)	*P* = 0.08
Wound dehiscence	0%	0.8% (*n* = 1)	*P* = 0.16
Overall complication rate	3.4% (*n* = 3)	7.6% (*n* = 9)	*P* = 0.09

*No statistically significant difference between the complication rates of the two groups was found.*

Complications developed at a median of 17 months following the operation, with only 42% of the complications (5 cases) developing during the first year postoperatively. There was no significant difference between the complication rates of each individual surgeon. The most frequent complication was urethrocutaneous fistula, reported in six patients. Of the six patients who developed a urethrocutaneous fistula, four underwent a two-stage repair of the hypospadias. The second most frequent complication was meatal stenosis, which was reported in three patients. Of the three patients who developed meatal stenosis, two underwent a two-stage repair of the hypospadias. Two patients who had both undergone a two-stage repair developed a urethral stricture, and in one patient who had also undergone a two-stage repair, wound dehiscence was observed 14 days after the operation. No statistical significance between the individual complication rates in the two groups was observed (Table 1). No further complications such as diverticulum, glans dehiscence, hair or stones in the urethra were reported in our study.

All the patients that developed complications in our study, with the exception of the patient with a wound dehiscence 14 days following the urethroplasty, underwent by reoperation a diagnostic cystoscopy to evaluate the neourethra. However, no cases of urethrocutaneous fistula following obstructive stricture of the urethra or the neomeatus were observed in our study. All of the five patients that developed meatal stenosis and urethral stricture in our study hat obstructive urinary symptoms and in three of the five patients, that were toilet trained at the time the complication manifested, a pathological uroflowmetry (Qmax < 7 ml/s) was documented. In two of the five patients with meatal stenosis or urethral stricture, episodes of lower urinary tract infection were also reported.

## Discussion

Not all non-proximal hypospadias are alike, and the variability of anatomical features plays an important role in the selection process among the different surgical approaches (3). Urethral plate characteristics, such as the meatal position and diameter as well as the urethral plate width, have been repeatedly reported as independent risk factors for the development of complications following hypospadias repair (4–7).

The Thiersch-Duplay technique is an easy technique that can be applied without concern in cases of non-proximal hypospadias with a deep glanular groove. Numerous modifications of the technique for universal implementation in hypospadias cases without a deep urethral groove have been proposed. In 1997, Stock and Hanna (9) reported the “distal urethroplasty and glanuloplasty procedure” (DUG), combining a Heineke-Mikulicz meatoplasty with the Thiersch-Duplay technique, as a single-stage procedure for the repair of all glanular, coronal, or subcoronal hypospadias. In this study, an astounding overall complication rate of 2.1% was reported (9). Unfortunately, no data concerning the length of the follow-up were provided in the publication. In 2018, a similar but much smaller study confirmed the excellent results of the DUG procedure in all types of glanular and coronal/subcoronal hypospadias (15). The study reported the occurrence of complications in only one out of 24 patients, equating to a complication rate of 4.2%.

The most popular modification of the Thiersch-Duplay procedure that can be applied to all cases of non-proximal hypospadias is the tubularization of an incised urethral plate (tubularized incised plate—TIP technique). Introduced by Snodgrass in 1994 (10), it has since gained widespread acceptance as a single-stage operation procedure for the repair of non-proximal hypospadias. The TIP repair technique and its modifications have been demonstrated in numerous studies to deliver excellent cosmetical and functional results (11, 16). However, discussion on whether the incision of the urethral plate performed immediately before urethral plate tubularization may lead to excessive scarring and consecutive obstruction has emerged repeatedly over time (17, 18). Extended transsection of the urethra and the paraurethral tissue performed during TIP, followed immediately by tubularization and formation of the neo-urethra, remains controversial, and can, according to some studies, lead to vascularization problems and may be responsible for stricture formation (19), which consecutively may lead to the formation of a urethrocutaneous fistula (17). In the formation of the neourethra, only healthy, well-vascularized tissue should be used (20). A large, randomized, prospective study from 2016 comparing the TIP technique vs. the tubularization of an intact and laterally augmented plate came to the clear conclusion that the tubularization of an intact urethral plate was significantly related to lower complication rates (21). The preservation of the underlying fascia combined with tensionless suturing of the neourethra, which is entirely lined with an intact epithelial layer, is believed to lead to reduced scarring of the neourethra and has been proven to be associated with lower complication rates (21). On the other hand, a series of studies has reported no correlation of urethral plate characteristics with urethroplasty complications when the TIP repair technique was applied (11).

**FIGURE 4:**
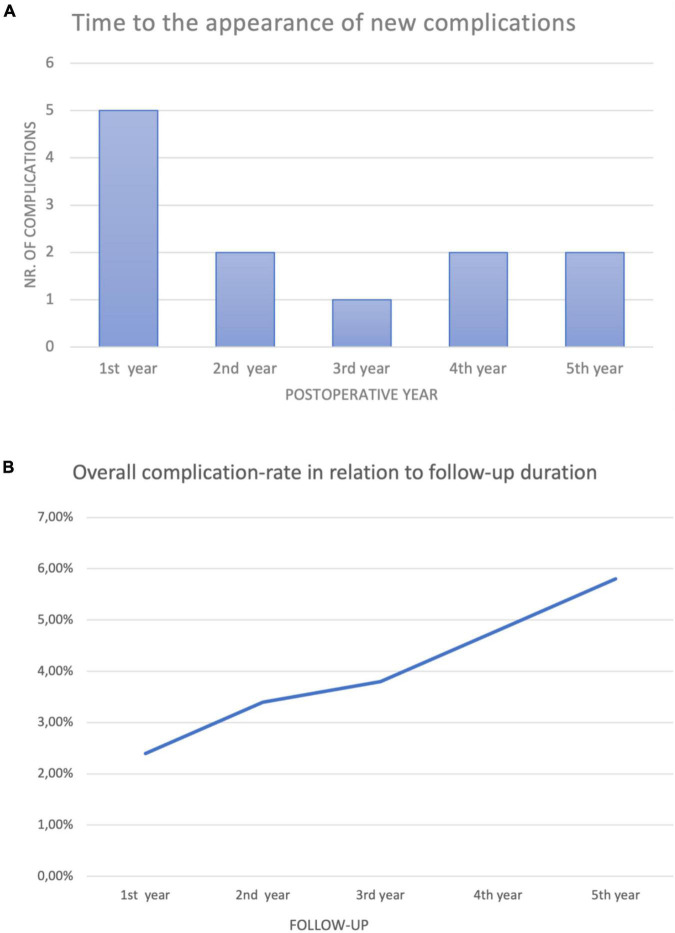
**(A)** Only five of the 12 complications reported (42%) presented within the first year following the repair. At a median follow-up of 50 months, complications in our study were reported as late as 53 months following primary hypospadias repair. **(B)** Continuous follow-up of the patients increased the overall complication rate. The duration of follow-up is essential in comparing surgical techniques for the repair of non-proximal hypospadias.

Although good results using single-stage procedures for the primary repair of all non-proximal hypospadias have been reported, our study shows that anatomically complex cases repaired in two stages are not associated with a higher rate of complications. Although the complication rates in our study were slightly higher in comparison to some of the abovementioned reports, we believe that this difference reflects the prolonged follow-up of our study patients, which we find is essential for truly evaluating hypospadias repair techniques. Despite the fact that some studies have claimed that 81% of urethroplasty complications can be detected within 1 year of primary hypospadias repair (22), the results of our study have shown that only 42% of the complications developed during the first year, with some complications in our study developing as late as four and a half years following the repair (with a median follow-up of 50 months) (Figure 4). Large retrospective studies have confirmed that late complications are not as rare as initially believed (23, 24).

Nevertheless, the complication rates in our study remain far below the average complication rates of non-proximal hypospadias repair in children. In particular, the systematic meta-analysis of published studies of non-proximal hypospadias repair techniques revealed an overall complication rate of approximately 8% (25). Urethrocutaneous fistula was, as expected, the primary complication reported, with an incidence of 4%, followed by meatal stenosis with 2.1% and urethral stricture with 0.8%. The review confirmed once again that the most popular technique currently used is the TIP technique. Snodgrass himself called TIP hypospadias repair an indicator operation in pediatric urology (11). A meta-analysis of complication rates of TIP repair, when applied to primary distal hypospadias, showed complication rates of 5.7% for fistula, 3.6% for meatal stenosis and 1.3% for urethral stricture (26). This 2015 meta-analysis of the TIP hypospadias repair technique reported limited documentation of follow-up in the included studies. In particular, 10 of the 49 studies included gave no length of follow-up, 14 reported a follow-up of less than 12 months, and 17 gave a follow-up duration between 12 and 24 months.

Undoubtedly, the greatest weakness of our study is the need for a two-stage repair for anatomically complex cases with adverse urethral characteristics. Apart from the risk of a second general anesthesia, two-stage repairs are associated with substantial inconvenience for the patients and their caregivers and can have an important negative psychological impact on the boys, which can be carried with them later on in adult life (27). However, it is our opinion, that a possibly reduced complication risk for anatomically complex cases is fair justification for a two-stage repair. Complications following hypospadias repair can be extremely traumatic, both to young patients and to their caregivers, with numerous reoperations often required (28). We have given great care in our center to perform hypospadias repair at an optimal timing around the age of 18 months (29), before children become aware of their genitalia, with the hope of reducing the perioperative stress as much as possible. However, this has not always been possible, as many of the patients were initially presented for repair at an older age.

In a systematic review from 2021 (3), it was demonstrated that urethral plate quality is an independent factor influencing postoperative outcomes of hypospadias repair, with only three studies in the review being larger than our study. Our study demonstrated, that following a two-stage surgical repair of patients with poor quality urethral characteristics, despite a larger number of patients and a longer follow-up as most of the abovementioned studies, complications were not statistically higher, when compared to patients with ideal urethral characteristics. Although complications were numerically higher in the two-stage group and even if the argument, that perhaps our study sample was not large enough for the statistical difference to become significant is correct, results of our study suggest that a two-stage repair of patients with adverse urethral characteristics may be associated with reduced complications. The main limitations of our study are its retrospective design and the lack of objective criteria regarding the urethral plate, introduced in recent years, such as the POST (Plate Objective Scoring Tool) (30) or GMS-score (Glans-Urethral Meatus-Shaft score) (31), as well as objective criteria regarding the cosmetic appearance of the penis following hypospadias repair, such as the HOPE-score (Hypospadias Objective Penile Evaluation-score) (32). Furthermore, the complete evaluation of a surgical procedure should involve long-term follow-up of the patients (33), well into adulthood, and ideally incorporate information regarding the psychosexual outcome, sexual function, and long-term satisfaction of the patients (34). It should remain clear that our study is a retrospective evaluation of the Thiersch-Duplay technique alone and results may differ when other repair techniques are applied. We believe that large, prospective, multicenter studies with objective criteria for the classification of the urethral plate and the evaluation of the cosmetic outcome, as well as a long-term follow-up of the patients, are needed to finally answer the question of which hypospadias repair technique is best suited or if a two-stage repair should be applied, depending on the individual anatomical variations of each patient. Hypospadias repair techniques should not be forced on the patient, but the procedure should be adapted to the specific anatomy encountered.

## Conclusion

Urethral plate characteristics, such as the meatal position and diameter as well as the width of the urethral plate, have been repeatedly reported as independent factors for the development of complications following non-proximal hypospadias repair. Our study shows that patients with distal hypospadias and poor urethral plate characteristics repaired in a two-stage approach have comparable low-complications to those with favorable urethral plate characteristics repaired in a single-stage. Furthermore, our study confirms the low complication rates of the Thiersch-Duplay urethroplasty and glanuloplasty procedure when compared with the average complication rates of other non-proximal hypospadias repair techniques.

## Data Availability Statement

The raw data supporting the conclusions of this article will be made available by the authors, without undue reservation.

## Ethics Statement

Ethical review and approval was not required for the study on human participants in accordance with the local legislation and institutional requirements. Written informed consent from the participants’ legal guardian/next of kin was not required to participate in this study in accordance with the national legislation and the institutional requirements.

## Author Contributions

MM and KH-K: project development, data collection, data analysis, manuscript writing, and manuscript editing. S-MB: data collection and data analysis. BW: project development, data analysis, manuscript writing, and manuscript editing. All authors have made substantial contributions to the material submitted for publication, all have read and approved the manuscript, and they have no commercial financial incentive associated with publishing the article.

## Conflict of Interest

The authors declare that the research was conducted in the absence of any commercial or financial relationships that could be construed as a potential conflict of interest.

## Publisher’s Note

All claims expressed in this article are solely those of the authors and do not necessarily represent those of their affiliated organizations, or those of the publisher, the editors and the reviewers. Any product that may be evaluated in this article, or claim that may be made by its manufacturer, is not guaranteed or endorsed by the publisher.
